# Molecular Detection of *Anaplasma phagocytophilum* and *Ehrlichia* Species in Ticks Removed from Humans in the Republic of Korea

**DOI:** 10.3390/microorganisms10061224

**Published:** 2022-06-15

**Authors:** Yu-Jung Kim, Ji Ye Seo, Seong Yoon Kim, Hee Il Lee

**Affiliations:** Division of Vectors and Parasitic Diseases, Korea Disease Control and Prevention Agency, 187 Osongsaenmyeong 2-ro, Osong-eup, Heungdeok-gu, Cheongju 28159, Chungbuk, Korea; hoiyui@korea.kr (Y.-J.K.); seojiye02@korea.kr (J.Y.S.); gunbo0402@korea.kr (S.Y.K.)

**Keywords:** *Anaplasma phagocytophilum*, *Ehrlichia* sp., ticks, human granulocytic anaplasmosis (HGA), human monocytic ehrlichiosis (HME), Republic of Korea

## Abstract

Human granulocytic anaplasmosis (HGA) and human monocytic ehrlichiosis (HME) are zoonotic tick-borne diseases transmitted via tick bites. To determine the state of human *Anaplasma* and *Ehrlichia* infections caused by tick bites in the Republic of Korea (ROK), we conducted a nationwide investigation of human cases of tick bites in 2020. A total of 180 ticks were obtained, comprising *Haemaphysalis longicornis* (70.0%), *Amblyomma testudinarium* (17.8%), *Ixodes nipponensis* (6.1%), *H. flava* (4.4%), and *I. persulcatus* (1.7%). In three cases (1.7%; 95% CI: 0.3–4.9), *A. phagocytophilum* was detected in *Ixodes* ticks using primers for *Anaplasma*-specific genes (16s rRNA, *ank*A, and *msp*4). Conversely, *Ehrlichia* sp. was only detected in *H. longicornis*, in two cases (1.1%; 95% CI: 0.1–4.0). To the best of our knowledge, this is the first record of *Ehrlichia* sp. in ticks parasitizing humans in the ROK. As concerns remain about the possibility of HGA and HME transmission, continuous monitoring and management of the pathogens and vectors are necessary.

## 1. Introduction

Ticks are major arthropod vectors of various pathogens, such as protozoa, bacteria, viruses, and parasites, which cause diseases in humans and livestock [1]. Under natural conditions, numerous tick-borne pathogens (TBPs) circulate between animals and ticks [2]. When acquiring a blood meal, ticks can transmit pathogenic organisms [3], such as *Anaplasma*, *Ehrlichia* [4], *Rickettsia* [5], *Bartonella*, *Borrelia*, *Babesia* [6], and severe fever with thrombocytopenia syndrome virus [7], to the host.

Human granulocytic anaplasmosis (HGA) and human monocytic ehrlichiosis (HME) are emerging zoonotic diseases caused by *A. phagocytophilum* and *E. chaffeensis*, respectively, which belong to the family *Anaplasmataceae*. The major clinical signs and symptoms of HGA and HME are nonspecific, such as fever, myalgia, headache, thrombocytopenia, leukopenia, and elevated levels of hepatic enzymes [8,9].

Patients with HGA and HME were first reported in the US in 1994 and 1987, respectively [10,11], and the number of patients has increased every year since, according to data reported by the Centers for Disease Control and Prevention [12]. Additionally, cases have also been reported in Europe and Asia [13,14,15,16]. In the Republic of Korea (ROK), *A. phagocytophilum* and *E. chaffeensis* were first identified in 2002 in the sera of patients with acute febrile disease [17], and the first patients were reported in 2014 and 2000 [18,19], respectively. Since the Korea Disease Control and Prevention Agency (KDCA) initiated its investigation into the incidence of HGA in 2015, the number of patients has increased gradually, with 4 cases reported in 2016, 13 in 2017, 32 in 2018, 38 in 2019, and 31 in 2020 [20]. However, no case of HME has been reported since the first suspected case in 2000 [19].

HGA and HME are transmitted by *Ixodes* sp. (*I. scapularis*, *I. ricinus*, and *I. pacificus*) and *Amblyomma americanum* in the US and Europe [6,21]. In the ROK, *Haemaphysalis longicornis*, *I. nipponensis*, and *I. persulcatus* have been identified as the main vectors for these pathogens [4,22], and domestic and wild mammals are considered as reservoirs [23,24,25]. However, few studies on TBPs in ticks isolated from humans bitten by ticks have been reported in the ROK. Recently, 16 ticks collected between 2014 and 2017 from residents of the southwestern region of the ROK tested positive for *A. phagocytophilum* (three ticks), *Babesia gibsoni* (one tick), *B. microti* (two ticks), and *Rickettsia* spp. (12 ticks) [26], with *A. phagocytophilum* infection detected in both ticks and patients [27]. However, no research has been conducted based on nationwide surveys.

The emergence and spread of TBPs are increasing due to global warming and other factors, such as increased human travel, animal transport, and urban development [28]. Therefore, continuous surveillance is necessary for monitoring the emergence of human diseases caused by TBPs [29,30]. As a public service, KDCA conducts annual pathogen investigations on ticks that bite humans. In this study, the presence of *Anaplasma* and *Ehrlichia* was investigated in cases of human tick bites across the ROK in 2020.

## 2. Materials and Methods

### 2.1. Tick Collection and Identification

Ticks were collected from local public health centers in the ROK from March to October 2020 as part of a service provided by the KDCA for the diagnosis of TBP infections in humans with tick bites from whom ticks were removed. The tick species and developmental stages were classified based on morphological classification keys [31]. Individual ticks were then placed in 2.0 mL cryovials according to the species, date, and stage of development, and were stored at −80 °C until DNA extraction.

### 2.2. DNA Extraction

Each identified tick was individually homogenized mechanically using a Precellys Evolution homogenizer (Bertin Technologies, Bretonneux, France) with phosphate-buffered saline and 2.8 mm beads (30 frequencies/s for 2 min), and then centrifuged at 12,000× *g* for 10 min at 4 °C. Following centrifugation, genomic DNA was harvested with the MagMAX™ DNA Multi-Sample Ultra 2.0 Kit (Applied Biosystems, Waltham, MA, USA) using the KingFisher Flex system (ThermoFisher Scientific, Waltham, MA, USA), according to the manufacturer’s instructions. The extracted DNA was stored at −20 °C until use.

### 2.3. Polymerase Chain Reaction (PCR) Amplification

Conventional PCR was performed using primers targeting the 16S rRNA gene sequence for each *Anaplasma* sp. and *Ehrlichia* sp., and nested PCR was performed using genospecies-specific primers against *ank*A, *msp*4, and *gro*EL, as described in previous studies (Table 1). Total genomic DNA of laboratory strains of *A. phagocytophilum* and *E. chaffeensis*, provided by the Division of Zoonotic and Vector Borne Disease Research, and the Division of Bacterial Diseases, KDCA, respectively, served as the positive control. Conventional and nested PCRs were performed in a total reaction volume of 20 µL. Each PCR mixture contained AccuPower^®^ PCR PreMix (Bioneer, Seoul, Korea), 10 pmol of each primer, 5 µL of DNA extracted from the ticks for the primary PCR, and 1 µL of the first-step PCR product used as a template for nested PCR. Each reaction was conducted in a C1000 Touch Thermal Cycler (Bio-Rad Laboratories, Hercules, CA, USA), as described in Table 1. The PCR products were visualized using gel electrophoresis in 1.2% agarose gel containing 10,000× Safe-Pinky DNA Gel Staining Solution (GenDEPOT, Barker, TX, USA). To avoid cross contamination, DNA extraction, amplification, and agarose gel electrophoresis were performed in separate rooms.

### 2.4. Nucleotide Sequencing and Phylogenetic Analysis

The PCR products that exhibited positive bands were subjected to sequencing at BIOFACT (Daejeon, Korea). To typify the isolates, the obtained sequences were matched against the National Center for Biotechnology Information (NCBI) nucleotide collection using the BLAST service, and aligned using CLUSTAL Omega (v.1.2.1). A phylogenetic tree was generated using the neighbor-joining method and the Kimura 2-parameter distance model in the MEGA 5.2 program. For assessing the bootstrap values of the obtained tree, 1000 bootstrap replicates were obtained.

## 3. Results

### 3.1. Identification of Ticks

A total of 180 ticks, including five tick species belonging to three genera, were collected from local public health centers in 2020. Among them, *H. longicornis* was the most abundant species (*n* = 126, 70%), followed by *A. testudinarium* (*n* = 32, 17.8%), *I. nipponensis* (*n* = 11, 6.1%), *H. flava* (*n* = 8, 4.4%), and *I. persulcatus* (*n* = 3, 1.7%) (Table 2 and Table 3). Based on the developmental stage, the 180 ticks comprised 110 adults (61.1%, 103 females and 7 males), 69 nymphs (38.3%), and one larva (0.6%) (Table 2 and Table 3). The collected ticks showed the highest prevalence between May and August (82.8%) (Table 2). The greatest number of ticks was collected from Gyeongsangbuk-do (*n* = 38, 21.1%), followed by Gyeongsangnam-do (*n* = 37, 20.6%), Gyeonggi-do (*n* = 33, 18.3%), and Chungcheongnam-do (*n* = 23, 12.8%) (Table 3).

### 3.2. Detection of Anaplasma *sp.* and Ehrlichia *sp.*

Based on the 16S rRNA gene analysis, out of 180 ticks, 3 ticks tested positive for *Anaplasma* sp. (1.7%; 95% CI: 0.3–4.9) and 2 ticks tested positive for *Ehrlichia* sp. (1.1%; 95% CI: 0.1–4.0). No coinfection was observed between the target pathogens. Based on the results of genospecies-specific nested PCR, three ticks tested positive for *ank*A and *msp*4 gene fragments (381 and 664 bp, respectively) of *A. phagocytophilum*, and two ticks tested positive for the *gro*EL gene fragment (365 bp) of *Ehrlichia* sp. Two *I. nipponensis* ticks and one *I. persulcatus* tick tested positive for *A. phagocytophilum*, whereas only one *H. longicornis* tick tested positive for *Ehrlichia* sp. All ticks that tested positive for the pathogens were matured females. The 16S rRNA gene and genospecies sequences detected in this study have been submitted to GenBank (accession numbers: OM681329-OM681333 and OM294660-OM294667).

### 3.3. Molecular and Phylogenetic Analysis

**Figure 1:**
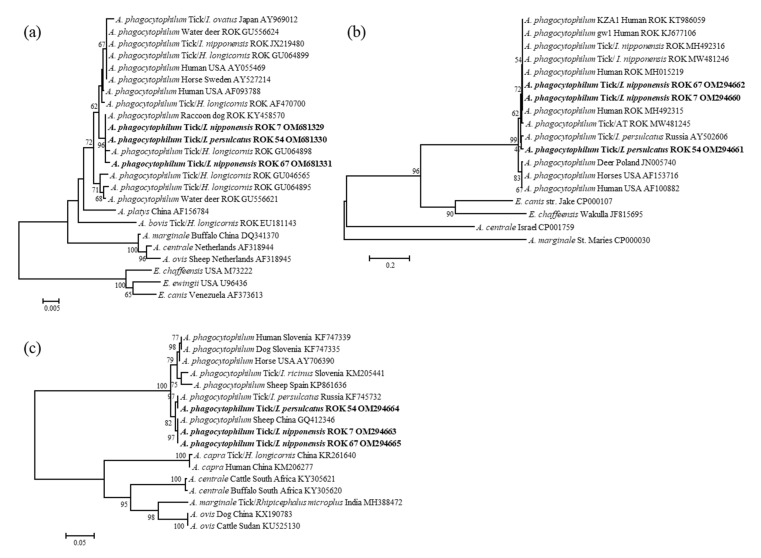
Phylogenetic relationships for *Anaplasma phagocytophilum*, based on the partial nucleotide sequence of (**a**) *Anaplasma* 16S rRNA, (**b**) *ank*A, and (**c**) *msp*4 gene. The neighbor-joining method was used for constructing a phylogenetic tree. The numbers at the nodes represent the proportion of bootstrap values for the branch point. The three *A. phagocytophilum*-positive sequences identified in this study are indicated in bold. Reference strains of *Anaplasma* with the host, country of detection, and the National Center for Biotechnology Information accession numbers are also shown. Scale bars indicate sequence distances.

The *Anaplasma* sp.- and *Ehrlichia* sp.-positive sequences were obtained in partial and aligned with the homologous sequences from the NCBI GenBank nucleotide sequence database. The 16S rRNA gene analysis revealed that among the three *Anaplasma*-positive ticks, samples nos. 7 (OM681329) and 54 (OM681330) were identical to each other, and the sequence obtained exhibited 100% identity with that of *A. phagocytophilum* isolated from a raccoon dog in the ROK (KY458570). Additionally, the sequence from sample no. 67 (OM681331) shared 99.78% identity with that of *A. phagocytophilum* detected in a tick in the ROK (GU064898) (Figure 1a). Sequence alignment of *ank*A indicated that sample nos. 7, 67, and 54 shared 100% and 98.29% identity with *A. phagocytophilum* isolated from *I. nipponensis* in the ROK (MW481246) and *I. persulcatus* in Russia (AY502606), respectively (Figure 1b). The partial *ank*A sequences were grouped with those of *A. phagocytophilum* strains isolated from ticks and humans in the ROK. Sequence alignment of *msp*4 indicated that sample nos. 7, 67, and 54 shared 100% identity with *A. phagocytophilum* isolated from sheep in China (GQ412346) and a tick in Russia (KF745732) (Figure 1c).

**Figure 2:**
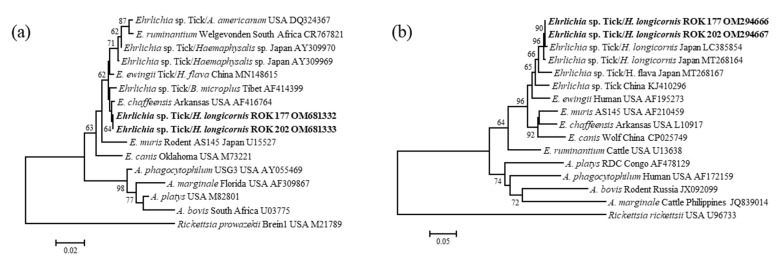
Phylogenetic relationships for *Ehrlichia* sp., based on the partial nucleotide sequence of (**a**) *Ehrlichia* 16S rRNA and (**b**) *gro*EL gene. The neighbor-joining method was used for constructing a phylogenetic tree. The numbers at the nodes represent the proportions of bootstrap values for the branch point. The two *Ehrlichia* sp.-positive sequences identified in this study are indicated in bold. Reference strains of *Ehrlichia* with the host, country of detection, and the National Center for Biotechnology Information accession numbers are also shown. Scale bars indicate sequence distances.

In the phylogenetic analysis of *Ehrlichia* species, the partial 16S rRNA gene sequences obtained in this study showed high identity (99.7%) with the sequences of *E. chaffeensis* isolated from the USA (AF416764) (Figure 2a). However, the partial *gro*EL sequences obtained from the two *Ehrlichia*-positive ticks showed 99.7% identity (99% coverage) with that of *Ehrlichia* sp. detected in *H. longicornis* in Japan (LC385854) and confirmed cluster formation with sequences of *Ehrlichia* sp. in ticks collected in Asia (Figure 2b).

## 4. Discussion

In this study, a total of 180 tick specimens were collected from humans during a nationwide investigation in the ROK, and molecular detection and phylogenetic analysis of three *A. phagocytophilum* and two *Ehrlichia* sp. pathogens were performed. Studies have been published on the molecular detection of TBPs in ticks that bite humans. Jahfari et al. [38] reported that several TBPs, including *Borrelia burgdorferi sensu lato*, *A. phagocytophilum*, *Candidatus Neoehrlichia mikurensis*, two *Rickettsia* species, and several *Babesia* species, in 314 ticks (removed from people with tick bites) and 626 blood samples (of people with tick bites or erythema migrans), were identified using PCR-based methods. Moreover, Xu et al. [39] investigated the infection prevalence of *B. burgdorferi sensu lato*, *B. miyamotoi*, and *A. phagocytophilum* in human-biting ticks collected over a 10-year period in three western states of the US [39]. However, studies on TBPs in the ROK have primarily been conducted on wild or domesticated animals, such as goats [23], deer [24], and cattle [25], and several sporadic cases have been reported in patients with tick bites or in individuals visiting local hospitals [26,27]. To the best of our knowledge, this is the first report of a nationwide survey to test ticks removed from humans in the ROK.

*H. longicornis* is most dominant tick species and is considered an important vector for tick-borne diseases in the ROK [5,40]. The present study showed that *H. longicornis* (70.0%) is the most common species detected in cases of human tick bites, followed by *A. testudinarium* (17.8%), *I. nipponensis* (6.1%), *H. flava* (4.4%), and *I. persulcatus* (1.7%). These findings are consistent with the results of a previous study in the ROK, which identified *H. longicornis* as the dominant questing tick species collected from various habitats [40,41]. In addition, previous studies have shown that *A. testudinarium* has a relatively low population density collected by dragging, flagging, and dry ice-baited trapping [7,42]. This species is known to use a host-seeking strategy, unlike the other ticks collected in this study that have a passive ambushing strategy [43]. Interestingly, in this study, the population density of *A. testudinarium* appeared to be relatively high compared to that reported in several other studies [42,44]. Recent studies conducted in the ROK have reported peaks in adult, nymph, and larval tick density from June to August, May to June, and August to September, respectively [41,45]. In this study, the monthly density of ticks at each developmental stage was similar to the results obtained from previous studies, except for tick larva (*n* = 1) in October. However, despite the epidemiological importance, the data did not provide estimates of the species composition and seasonal abundance of ixodid species removed from humans in the ROK. Nonetheless, the results can be useful in providing the basis for vector-borne risk assessments of tick bites.

*A. phagocytophilum* is the most frequently reported TBP in the ROK since the first reported case in 2002 [5,22]. In accordance with findings from previous studies, *A. phagocytophilum* has been detected in ticks feeding on livestock and wild animals, including cattle (31/566 tick pools (5.5%)) [46], Korean water deer (89/266 tick pools (33.5%)) [47], horses (5/1409 tick pools (0.4%)) [48], and migratory birds (1/108 tick pools (0.9%)) [49]. In a study, 1467 ticks were collected from nine provinces of the ROK, and 35 *H. longicornis* ticks and 1 *I. persulcatus* tick were found to test positive for *A. phagocytophilum* [4]. Various TBPs were found in 33 ticks isolated from humans in the southwestern region of the ROK between 2014 and 2017 [26]. Among them, 9.1% tested positive for *A. phagocytophilum* (two *I. nipponensis* ticks and one *A. testudinarium* tick). In this study, we surveyed TBPs in ticks removed from humans bitten by ticks throughout the country, and *A. phagocytophilum* was detected in two *I. nipponensis* ticks and one *I. persulcatus* tick. Each 16S rRNA, *ank*A, and *msp*4 gene fragment obtained from *A. phagocytophilum*-positive ticks formed a cluster with the corresponding sequences of *A. phagocytophilum* identified in ticks or in animals and Korean patients.

*E. chaffeensis* is the etiological agent of HME [50] and has been primarily detected in *Ixodes* sp. in the US and Europe [6,21]. In the ROK, *E. chaffeensis* is most frequently detected in *H. longicornis* ticks collected from the Gyeonggi province (4.3%, 26/611 ticks) [36], the Korean Demilitarized Zone (15.0%, 63/420 tick pools) [51], and Jeju Island (12.1%, 56/463 salivary glands) [52]. In this study, *E. chaffeensis* was not detected; instead, two *H. longicornis* ticks from Gyeonggi and Gyeongsangnam provinces tested positive for *Ehrlichia* sp., with unknown pathogenicity to humans. According to Kim et al. [4], *Anaplasma* and *Ehrlichia* sp. are extensively distributed across the ROK [4]. Phylogenetic analysis based on the 16S rRNA and *gro*EL gene sequences of *Ehrlichia* species revealed different results. In general, 16S rRNA gene amplification has been used for the identification of bacterial pathogens [53]. However, evident sequence comparison is limited owing to high conservation. *gro*EL sequences are more divergent than the corresponding 16S rRNA gene sequences, and are considered a valuable tool for phylogenetic analysis [54]. For this reason, analysis based on *gro*EL gene sequencing is more reliable. In this study, two samples belonging to the genus *Ehrlichia* were found to be more closely related to *Ehrlichia* sp. with unclear characteristics for pathogenicity as isolated from *H. longicornis* in Japan [55] than *E. chaffeensis*. To the best of our knowledge, this is the first report on the presence of *Ehrlichia* sp. in ticks removed from humans in the ROK.

Climate patterns are changing rapidly owing to global warming, and the range of tick habitats is spreading widely; hence, various tick-borne diseases are expected to emerge and re-emerge [56,57]. In addition, as outdoor activities such as climbing and camping increase, the probability of human contact with ticks increases, which is expected to pose a threat to public health. Therefore, the continuous monitoring of various tick species and hosts and corresponding preventive measures are necessary.

## Figures and Tables

**Table 1:** Primers used for the detection of *Anaplasma* and *Ehrlichia* in ticks.

Target Gene	Primers		Sequence (5′ to 3′)	Amplicon Size (bp)	PCR Conditions	References
*Anaplasma* 16s rRNA	EE1	1st	TCCTGGCTCAGAACGAACGCTGGCGGC	1433	94 °C/5 min; 35 cycles: 94 °C/60 s, 50 °C/30 s, 72 °C/1.5 min; 72 °C/10 min	[32]
	EE2		AGTCACTGACCCAACCTTAAATGGCTG			
	EE3	2nd	GTCGAACGGATTATTCTTTATAGCTTGC	926	94 °C/5 min; 35 cycles: 94 °C/30 s, 50 °C/30 s, 72 °C/60 s; 72 °C/10 min	
	EE4		CCCTTCCGTTAAGAAGGATCTAATCTCC			
*Anaplasma ank*A	ANK-F1	1st	GAAGAAATTACAACTCCTGAAG	705	94 ℃/2 min; 40 cycles: 94 °C/30 s, 55 °C/30 s, 72 ℃/60 s; 72 °C/5 min	[33]
	ANK-R1		CAGCCAGATGCAGTAACGTG			
	ANK-F2	2nd	TTGACCGCTGAAGCACTAAC	664	94 °C/2 min; 30 cycles: 94 °C/30 s, 55 °C/30 s, 72 °C/60 s; 72 °C/5 min	
	ANK-R2		ACCATTTGCTTCTTGAGGAG			
*Anaplasma* *msp*4	MSP4AP5	1st	ATGAATTACAGAGAATTGCTTGTAGG	849	94 °C/5 min; 35 cycles: 94 °C/60 s, 54 °C/60 s, 72 °C/60 s; 72 °C/10 min	[34]
	MSP4AP3		TTAATTGAAAGCAAATCTTGCTCCTATG			
	MSP4f	2nd	CTATTGGYGGNGCYAGAGT	381	94 °C/5 min; 30 cycles: 94 °C/30 s, 55 °C/30 s, 72 °C/30 s; 72 °C/10 min	
	MSP4r		GTTCATCGAAAATTCCGTGGTA			
*Ehrlichia* 16s rRNA	AE1-F	1st	AAGCTTAACACATGCAAGTCGAA	1406	94 °C/5 min; 40 cycles: 94 °C/60 s, 59 °C/60 s, 72 °C/1.5 min; 72 °C/10 min	[35]
	AE1-R		AGTCACTGACCCAACCTTAAATG			
	HE1	2nd	CAATTGCTTATAACCTTTTGGTTATAAAT	390	94 °C/3 min; 3 cycles: 94 °C/60 s, 55 °C/2 min, 72 °C/1.5 min; 92 °C/60 s; 37 cycles: 92 °C/60 s, 55 °C/2 min, 72 °C/60 s; 72 °C/10 min	[36]
	HE3		TATAGGTACCGTCATTATCTTCCCTAT			
*Ehrlichia* *gro*EL	GR0607F	1st	GAAGATGCWGTWGGWTGTACKGC	664	95 °C/5 min; 35 cycles: 95 °C/30 s, 54 °C/30 s, 72 °C/60 s; 72 °C/10 min	[37]
	GR01294R		AGMGCTTCWCCTTCWACRTCYTC			
	GR0677F	2nd	ATTACTCAGAGTGCTTCTCARTG	315	95 °C/5 min; 30 cycles: 94 °C/30 s, 57 °C/30 s, 72 °C/60 s; 72 °C/10 min	
	GR01121R		TGCATACCRTCAGTYTTTTCAAC			

**Table 2:** Seasonal distribution of human-biting ticks and pathogen prevalence in the Republic of Korea, March–October, 2020.

Species	Stage	No. of Collected Ticks									*Anaplasma* *phagocytophilum*		*Ehrlichia* sp.	
		March	April	May	June	July	August	September	October	Total (%)	Positive (%)	95% CI	Positive (%)	95% CI
*Amblyomma* *testudinarium*	Female	-	-	1	5	1	1	2	1	11 (6.1)	0	0	0	0
	Larva	-	-	-	-	-	-	-	1	1 (0.6)	0	0	0	0
	Male	-	-	1	1	-	-	-	1	3 (1.7)	0	0	0	0
	Nymph	-	2	3	4	6	1	-	1	17 (9.4)	0	0	0	0
*Haemaphysalis* *flava*	Female	-	-	-	-	-	-	-	2	2 (1.1)	0	0	0	0
	Male	-	-	1	-	-	-	-	1	2 (1.1)	0	0	0	0
	Nymph	-	2	2	-	-	-	-	-	4 (2.2)	0	0	0	0
*H. longicornis*	Female	1	-	7	6	21	34 (2 ^‡^)	8	-	77 (42.8)	0	0	2 (2.6)	0.3–9.3
	Male	-	-	-	1	-	1	-	-	2 (1.1)	0	0	0	0
	Nymph	-	3	17	8	11	3	4	1	47 (26.1)	0	0	0	0
*Ixodes* *nipponensis*	Female	-	1 (1 ^†^)	4	4 (1 ^†^)	1	-	-	-	10 (5.6)	2 (20.0)	2.4–72.3	0	0
	Nymph	-	-	1	-	-	-	-	-	1 (0.6)	0	0	0	0
*I. persulcatus*	Female	-	-	3 (1 ^†^)	-	-	-	-	-	3 (1.7)	1 (33.3)	0.8–185.7	0	0
Total	Female	1	1	15	15	23	35	10	3	103 (57.2)	3 (2.9)	0.6–8.5	2 (1.9)	0.2–7.0
	Larva	-	-	-	-	-	-	-	1	1 (0.6)	0	0	0	0
	Male	-	-	2	2	-	1	-	2	7 (3.9)	0	0	0	0
	Nymph	-	7	23	12	17	4	4	2	69 (38.3)	0	0	0	0
	Total	1	8	40	29	40	40	14	8	180 (100.0)	3 (1.7)	0.3–4.9	2 (1.1)	0.1–4.0

^†^: positive for *A. phagocytophilum*, ^‡^: positive for *Ehrlichia* sp.

**Table 3:** Geographical distribution of human-biting ticks and pathogen prevalence as recorded in 2020 across the 14 administrative units of the Republic of Korea.

Region	Species					Total (%)
	*Amblyomma* *testudinarium*	*Haemaphysalis* *flava*	*Haemaphysalis* *longicornis*	*Ixodes* *nipponensis*	*Ixodes* *persulcatus*	
Seoul Special City	1		1			2 (1.1)
Gyeonggi-do Province	0	2	29	2		33 (18.3)
Gwangwon-do Province	0		5		2	7 (3.9)
Chungcheongbuk-do Province	0		9	1	1	11 (6.1)
Chungcheongnam-do Province	2		16	5		23 (12.8)
Jeollanam-do Province	2		4			6 (3.3)
Gyeongsangbuk-do Province	5	1	31	1		38 (21.1)
Gyeongsangnam-do Province	18	3	16			37 (20.6)
Jeju special self-governing Province	0		2			2 (1.1)
Metropolitan area *	4	2	12	2		20 (11.1)
Unknown			1			1 (0.6)
Total	32	8	126	11	3	180 (100)

* Metropolitan area includes Busan, Daejeon, Incheon, Sejong, and Ulsan.

## Data Availability

Data supporting the conclusions of this article are included within the article. The newly generated sequences were submitted to the GenBank database under the accession numbers OM681329-OM681333 and OM294660-OM294667. The datasets used and/or analyzed during the present study are available from the corresponding author upon reasonable request.
